# Adrian James

**DOI:** 10.1192/bjb.2023.13

**Published:** 2023-06

**Authors:** Abdi Sanati


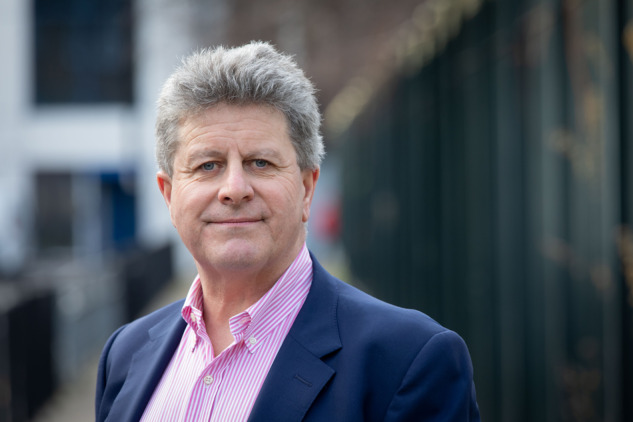


Dr Adrian James has been the President of the Royal College of Psychiatrist at one of the most difficult times for the College. The COVID-19 pandemic posed several challenges which I think the College managed well. I first met Dr James when I was a Regional Advisor and he had been appointed as the Registrar of the RCPsych. I was impressed by his ability to come up with a coherent synthesis of all the contradictory opinions that satisfied everyone. I learned a lot from observing his leadership and calmness during some very tough exchanges. This interview with him has been long overdue and I am glad I managed to do it before the end of his term as President.

**Many thanks Dr James for the interview. I remember in the hustings three years ago that you mentioned that you had many experiences – of being a psychiatrist, being a patient, being Registrar of the College – and you wanted to have the experience of the College's presidency. Now that you have had that experience what do you think about it?**

It has been professionally the most wonderful experience of my life by far and away. I'm very lucky that I still see patients. And so I actually think I get more fulfilment out of seeing patients now at the end of my career than I did even at the beginning. So I'm very lucky that I've kept that clinical base, but I would say that in terms of a role, and the opportunity to learn, it's the professional experience where I think I've learned the most, and it gives you such an opportunity to make a real difference for patients. I've worked with the most amazing people. I remember vividly, people would want to speak to you as the President, but if you can give them some of your time, individually, people will tell you their story. That has been such a learning experience for me, and to be able to take from those stories and say, I have an awareness of the system, I have contacts and I can do something with somebody's story to make somebody else's story different. It has probably been the most challenging. It's been the busiest and it has an impact on you and I don't think anybody should do it for more than three years. But I have really enjoyed it, even the things that people might look externally and think that must have been really tough. The tough things, I think, as an experience, have in many ways been the best ones.

**You become President during the pandemic and lockdown. You had to make some very hard decisions. Which were the most difficult?**

I felt that the College needed to provide some stability and familiarity in people's lives as psychiatrists. So I spoke to nearly every single department in the College and said we have to find a way of functioning and of reaching out and that was very challenging. There were people who came to me and said ‘I just don't think we can do this thing anymore’, and I said we really need to find a way because there's a need out there. The need is primarily for our patients but also working through our members. They're providing the service for patients, and that need is not going to go away. And so the service that we provide for them is something that we must keep. In terms of difficult decisions, I think some of them were really taken out of your hands. When the lockdown was there, we had to close the College building. That was very difficult. It was very difficult for our staff and members who couldn't access the College. But we did so much to put things online. We had the Thursday afternoon webinars. Lots of people came to me and said that they found that valuable; in fact in some ways they felt even more connected with the College. They were, in the early days, mostly free. All the ones about COVID were always free. But obviously we have an obligation to keep the College functioning. So we have to earn money in order to do that. But it was something which was very traumatic for people. And the impact on our patients' and psychiatrists' lives was absolutely huge.

**I have to admit I was worried about how the College would survive. I was quite amazed when I saw the College's balance sheet at the end of 2020, that the College was financially better off at the end.**

That's right. Because of the way in which we reached out to services. For example, our quality networks, where we have 1500 teams. We switched them to be quality networks based around COVID so they were giving people information. So for me, I work in a low secure unit – that's a low secure quality network. We switched it so that the network became a learning exercise about COVID. There was a thirst for information. This was all completely new. And we had a way of bringing it together and then delivering to our members for the benefit of patients. I think that was an extraordinary thing – we didn't lose services, in fact, I think the numbers even went up.

Regarding our exams, that was a great triumph because although it was always our intention to digitise the written exams we never had any intention to digitise the clinical exam, and to do that within six months, and to keep that functioning was a priority for us. Again, it's ultimately about patient care and career progression and we wanted to make sure, however long the pandemic went on, that the trainees could continue to progress with their careers. So that was a huge achievement, I think. That cost us a lot of money but we weren't afraid to spend money. But of course then we have people taking the exam, so we were able to keep afloat. We also changed our investments to be green investments. We disinvested from fossil fuels, and green investments went up hugely. We didn't do it to make money. We did it, I think, for the right reasons, but sometimes it's nice in life, when you do things for the right reason and you get an extra bonus.

**The College has become very much online. And what do you think of that? I understand that some members are happier. And interestingly, in some of the meetings, I realised the attendance has gone up.**

Yes. We had 1000 psychiatrists attending some of our webinars. It was quite extraordinary. For the International Congress we had a bespoke online platform. We had a huge attendance online. And so at meetings as well, because people didn't have to travel to London, we found that it was easier to get everybody around the virtual tables. There were people who always found it more challenging to connect with the College – particularly people living in remote areas, people with caring responsibilities and people with disabilities. Suddenly they could connect as easily as everybody else. So they were a group of people who we were able to attract, but of course, now we're going back to having more face to face. I think it's a creative tension between saying, isn't it great to meet together, we must have our meetings together, we must have our international congress together and others saying – well, we feel shut out of that, so we would prefer online. I think in the end, there's got to be a mixture.

**There's one question I always wanted to ask. In all these years I've been a psychiatrist, I have seen many College presidents and have enormous respect for them. I have learned a lot from each president I have encountered. But one dominant question on my mind is, what the president can actually do? I am aware that the office comes with its own gravitas, social capital and connections. But what executive powers does the president possess?**

Think of any president of any medical Royal College. In many ways the president of our College has the most power, but you are also rightly constrained and there are mechanisms within the College that constrain an individual. I'm, first of all, elected by all the members. I think that gives you a particular position and gives you some credibility. It gives you more of a mandate, whereas for certain medical Royal Colleges, they might just be elected by their council. So I think the fact that the members ultimately say ‘We want this person’, it gives you an inherent sort of power. The President also chairs the Council and the Trustee Board, the two most important decision-making bodies. Again in other Colleges, sometimes the president does not chair those bodies. I also line manage the Chief Executive. So the Chief Executive reports to the President and again, that doesn't happen in every College. So the President is in a very powerful position, but even if I feel very strongly about something I can't just say this is what we are going to do. There is a process with checks and balances. Ultimately, if Council say we don't want to do something, they are an elected body, elected by our members, and they can constrain me and say ‘Well, you thought that was a good idea, but we don't agree’. I might then have to come back or I might have to drop something. But then, ultimately, it goes to the Trustee Board for those big issues around the governance of the College, the identity of the College, and then in our College, it comes back to our members, who vote for the biggest decisions of all, such as who can vote in elections. For example, around votes for affiliates – I thought it was right, the Council thought it was right, the Trustee Board thought it was right. When it came to members, we had to get a two-thirds majority. So although most of our members agreed with it, in order to make that big change, it had to be overwhelming. So I think that's probably a good thing that there are mechanisms to control me. There are mechanisms that ensure my power comes from the members, but then you have to go back to the members for those big decisions, like the identity and the boundaries of the College. So I think it works quite well. I think you can't have somebody running the organisation who is entirely constrained, because nothing will ever happen. You never get any change. And of course, there are also other decisions I have to make on the hoof. There were lots of those in the pandemic. I still have to bring them back to council. Because council may say ‘Well, that was a good thing’. Or they might say ‘Okay, you're under pressure, but you got that seriously wrong’. So that's another way of constraining, although it's tempting to think I'd just love it if I could just do anything I wanted to and everybody says yes. We have these checks and balances for good reasons.

**So you can't do a Trump and issue an executive order!**

I suppose it can happen in some situations. I think anybody ultimately running an organisation, you have to allow them to make some decisions and then report back. But I think those big things, those things around boundaries, identity, you have to go back to the members.

**Psychiatrists work in organisations – how much impact do you think the President has on issues like psychiatrists' contracts or the design of services? – because these are things that are actually difficult to influence.**

So in terms of terms and conditions, we influence, for example, through our regional advisors, looking at job descriptions and approving them or not. In terms of the big issues around things like pay, we don't get involved with that. That's a union matter. I think if we stray into that, that would be a mistake. In terms of how you get real change, for example in the numbers of psychiatrists, that's got to be an absolute in terms of your job. As the President it is your job to continue to grow the profession. I guess you have a big role in doing that, because you have to endlessly talk about psychiatry, in the press and with ministers and policymakers about the importance of psychiatry. I generally start by talking about the difference we can make, so that people would know what difference we make in patients' lives. You have to promote psychiatry. So people think psychiatry is a good thing and there are some very good things you can do with psychiatry. Then you need to make the case for how you could do an even better job if you had more psychiatrists – I have to make that case to ministers and policymakers. Sometimes you have to make that case in the press because politicians listen to the press. So you have to go to the press with a story about why things are not as they should be and what could happen. Then you get the interest of ministers and they have to speak to you because you have connections. So you have to grow your connections with the press. We have a policy department and they're endlessly sifting the evidence about what's happening and what could happen. We present evidence and data and make sure our policy department functions in a way that the data give me an opportunity to have a convincing argument. For example, we had the senior team from NHS England come to dinner at the College. I invited the Chief Executive and the Chair, who both said yes, which I thought was pretty amazing. We invited Claire Murdoch, the senior responsible officer for mental health, as well. Then they asked if two other people could come – the Medical Director of NHS England and the Head of Strategy. So there we were, around our table in the College, with an opportunity to sell psychiatry, to make sure that people are aware that we are proper people doing a proper job, and there's a good evidence base. I always thought there was this myth about mental health that it is the bottomless pit of money and you just throw money at it, it all disappears. So I started by saying, look what we did with perinatal psychiatry. We've now got the map of England ‘green’ for both community services and in-patient services. Look at the differences made to patients. Look at liaison psychiatry. We've got 88% of hospitals with a core 24 service, look at the difference that makes to patients. My own specialty in forensic psychiatry has been transformed in my professional lifetime. Look what we're doing in general psychiatry, and if you give us more money, this is what we could do in this area. I think those sorts of things have contributed hugely to us getting the money for extra posts in psychiatry. So we, in the next year, are going to have extra training posts in psychiatry. Unless I and others had been arguing the case, we'd never have got that money. I think we're going to have the biggest uplift in terms of extra training numbers of any specialty. And that wouldn't happen unless people believed you and so it's that complex web of good stories, data, evidence and use of the press. Getting people's confidence so they can trust that you're not going to say one thing to one person and something else to another. That you're not going to overdo it. They're not going to find that you're talking a load of nonsense just to make your case. So you're credible, but also really importantly, you deliver. I think what we've really delivered on is 100% recruitment into psychiatry. That is an astonishing achievement. That adds to our credibility hugely.

**You mentioned recruitment of 100%. But one thing that came to my mind is the retention. I think when I was a trainee, many, many years ago, the College at that time said only 14% of psychiatrists retire in their job. I am sure you agree that we have to do something to retain psychiatrists as the situation does not seem to have improved much.**

Absolutely. To put all this effort into getting people into our fantastic specialty and then to lose them is absolutely shocking and it's a waste. It's a waste of their time as well. You would always expect to lose some, and some people will discover that psychiatry is not for them. It's important that people can move out of psychiatry and do something which better suits them. But for most people who leave, it's not because they inherently don't like it or they don't want to do it. It's a whole load of other factors to do with the stress of the job, of feeling unsupported or feeling unsafe, either physically or psychologically unsafe. It has nothing to do with the actual job that you and I do with the patient in front of us. It's all the other stuff. I think that is the thing that we have to move on to. I think Choose Psychiatry will evolve and we have been having discussions that we need to move it on to keeping people in psychiatry. One of the things I'm most proud of is the work that we've done on quality improvement, bringing quality improvement methodology into mental health when people said it wasn't really possible to do that. I said why don't we have a quality improvement initiative around workforce well-being. And so we did develop that, we looked at the evidence on workforce well-being and how that's linked to retention. And we started a quality improvement initiative. We recruited I think about 40 teams and the evidence, which is still not published, is that if you take a quality improvement approach to it (and often it is about making simple changes to people's working environment), morale goes up hugely. And retention goes up. So I think there are things we can do about it and we have to take an evidence-based approach. Often evidence doesn't lead to doing lots of fancy stuff, it leads to doing simple stuff, actually.

**That takes us to the issue of the membership. During the last Council election, less than 20% of people voted. Voter turnout is low and what do you think of the poor engagement of members in the elections?**

I think we have to do more to connect with our members and to make them feel that their voice is heard. They have the power and they should realise it's worth their while to exercise that power. We're in the middle of a presidential election. They have the power to decide who the next president is. And so my understanding is, in our elections we're in the low 20s in terms of the percentage turnout. If you compare with the other medical Royal Colleges, that's actually quite good. So it's a feature generally of membership organisations that the turnouts tend to be low. It'll be interesting to see the turnout for this presidential election because there have been so many issues that our members have engaged with. I always said it was a good thing people were saying these items that really mattered to them. I think there are a lot of issues. I think we have to make the College something that everybody feels that they have a say in and that they have a stake in what the College does. If they think that, then they will be more likely to vote and to decide who are they going to put in a position. If they think that doesn't actually make any difference then I guess people will feel, well it's a waste of time.

**When it comes to voter engagement there are two ways to look at it. One is that they are well satisfied, and when they look at the nominees they think ‘These all good so I do not need to vote’. Or they are so dissatisfied that they can't be bothered. I recently joked with one of the presidential nominees that it would be interesting to add the option of** ‘**none of the above’ to the ballot. Is it that by not voting, the members are saying** ‘**none of the above’ or** ‘**all of the above’?**

It is hard to determine. I think whatever the decision we need to encourage every single member who has a vote to exercise it. None of us should be complacent about democracy. I feel very strongly that people need to always exercise their democratic right. We clearly need to do more to engage with our members. One of the ways we do that is the membership survey and actually we get a positive feedback from these. The other thing is that there are people who are very much connected to the College and pay their subscription and work day in and day out with our patients, but do not have a vote. That was one of the things that I tried to get through and will probably come back to during the next AGM and we have to see. I think no taxation without representation. It is not just the people who can and do not vote. There are people who pay and want to vote and cannot vote and feel excluded.

**In your role I can see that you go abroad and connect with other institutions around the world. How do you compare the state of UK psychiatry with, for instance, USA or Europe?**

I think we have huge challenges. And it's great that we all want to do better. But it's a rather humbling experience going to different parts of the world. I think the NHS and the work that our psychiatrists do stands up really very well. When you go to another country, it gives the opportunity to put some balance in, because I spend a lot of my time saying things aren't good enough, we need more of this or we need more of that. When you compare with other parts of the world, we have specialties that other equivalent countries just don't have. So I think that's really quite humbling. Of course there are other issues. The US is extraordinary, I think they spend about three times the amount per head of the population on health, but the mental health services for people who need them the most are very poor. Really, I don't think anybody would say they're not, but of course there's tremendous learning from different parts of the world, people who have very low numbers of psychiatrists, the way in which they've used communities, community leaders, in order to raise the profile of mental health, to get people talking about mental health, to destigmatise mental illness and look at community support. I think we can learn a lot from that, at a time when we know that we can never fully meet the need. We have to turn to communities and say, how can we look at our communities? How can we get them more mental health aware? We can get everybody talking about mental health to get people to support each other. And so I think we can learn a lot, but generally speaking, we do pretty well.

